# Acute Hypoglycemia Induces Painful Neuropathy and the Treatment of Coenzyme Q10

**DOI:** 10.1155/2016/4593052

**Published:** 2015-12-28

**Authors:** Yan Ping Zhang, Shanshan Mei, Jinfeng Yang, Yiliam Rodriguez, Keith A. Candiotti

**Affiliations:** ^1^Department of Anesthesiology, Perioperative Medicine and Pain Management, University of Miami Miller School of Medicine, Miami, FL 33136, USA; ^2^Department of Obstetrics and Gynecology, Guangzhou Women and Children's Medical Center, Guangzhou 510120, China; ^3^Department of Anesthesiology, The Affiliated Cancer Hospital of Xiangya School Of Medicine, Central South University, Changsha, Hunan 410013, China

## Abstract

Diabetic neuropathic pain is reduced with tight glycemic control. However, strict control increases the risk of hypoglycemic episodes, which are themselves linked to painful neuropathy. This study explored the effects of hypoglycemia-related painful neuropathy. Pretreatment with coenzyme Q10 (CoQ10) was performed to explore the preventive effect of CoQ10 on hypoglycemia-related acute neuropathic pain. Two strains of mice were used and 1 unit/kg of insulin was given to induce hypoglycemia. Mechanical sensitivity of hindpaw withdrawal thresholds was measured using von Frey filaments. Blood glucose levels were clamped at normal levels by joint insulin and glucose injection to test whether insulin itself induced hypersensitivity. Results suggest that the increased mechanical sensitivity after insulin injection is related to decreased blood glucose levels. When blood glucose levels remained at a normal level by the linked administration of insulin and glucose, mice demonstrated no significant change in mechanical sensitivity. Pretreatment with CoQ10 prevented neuropathic pain and the expression of the stress factor c-Fos. These results support the concept that pain in the diabetic scenario can be the result of hypoglycemia and not insulin itself. Additionally, pretreatment with CoQ10 may be a potent preventive method for the development of neuropathic pain.

## 1. Introduction

Diabetic neuropathy is a leading complication of diabetes mellitus, resulting in significant morbidity and mortality. Although its exact pathogenesis is not fully understood, hyperglycemia does not appear to be the sole factor in the development of neuropathy in diabetic patients. Enigmatically, recent reports have described that long-term tight glycemic control may be a major risk factor for the development of diabetic neuropathy [1, 2]. Neuropathy secondary to rapid normalization of chronic hyperglycemia in the setting of poorly controlled diabetes is also emerging as a new disease entity classified as an iatrogenic complication [3]. Symptoms in these patients are typically consistent with a distal sensory polyneuropathy which is appearing shortly after the initiation of intensive glycemic control and is referred to as “insulin neuritis” or treatment-induced neuropathy and is characterized by acute, severe pain [1].

The pathophysiology and incidence of “insulin neuritis” are unclear. However, the parallel worsening of neuropathy and retinopathy from a rapid tightening of glycemic control [4, 5] suggests a common underlying pathophysiology. Hypoglycemia, a potentially devastating neuronal insult, is usually the result of attempting tight control of blood glucose levels with insulin or other hypoglycemic agents [1, 6]. Currently, the only available method for preventing this hypoglycemia-induced neuronal injury in the clinical setting is the delivery of glucose, a treatment that paradoxically may exacerbate the insult.

The objective of this present research was to study the molecular mechanisms of acute neuropathic pain induced by insulin and hypoglycemia in an animal model. The expression of c-Fos protooncogene, a marker of nociceptive-induced neuronal activity in the spinal cord [7, 8], was also determined. Additionally, the preventive effects of pretreatment with coenzyme Q10 (CoQ10) on hypoglycemia-induced neuropathic pain and stress-sensitive factor expression were explored.

## 2. Materials and Methods

### 2.1. Animal Preparation

All experiments were carried out following the guidelines and protocols of the Animal Care and Use Committee of the University of Miami, and the protocol was approved by the IACUC committee. C57BL/6J mice served as controls and CBA/CaJ mice, which develop diabetes spontaneously, functioned as the treatment group; they were both obtained from Jackson Laboratory (Bar Harbor, Maine, USA). All mice were approximately 12 to 14 weeks old which is comparable to young adult in humans. While CBA/CaJ mice spontaneously develop mild hyperglycemia, these mice had not yet developed peripheral neuropathy at the commencement of the study, as assessed by mechanical testing.

Mice were housed in groups of five in plastic cages with soft bedding and free access to food and water under a 12 h/12 h light-dark cycle (dark cycle: 7:00 pm–7:00 am). All animals were acclimated in their cages for one week before experiments were begun.

### 2.2. Blood Samples and Blood Glucose Measurement

Blood from animals for glucose measurement was obtained via a tail tip snip. During collection, the initial blood expressed was discarded and a subsequent sample was analyzed with OneTouch glucometer.

### 2.3. Hypoglycemia Induction

To examine the effects of acute insulin-induced hypoglycemia on mechanical sensitivity, 1 unit/kg of insulin (Novolin, Novo Nordisk, 2880 Bagsværd, Denmark) was injected intraperitoneally in the treatment group, while control animals received equal volumes of normal saline. Blood glucose levels and mechanical sensitivity were tested before injection and periodically throughout the study until blood glucose levels recovered to normal.

### 2.4. Blood Glucose Clamp

To determine whether insulin itself or insulin-induced hypoglycemia was the cause of mechanical hypersensitivity, blood glucose levels were “clamped” in the normal range by the combined administration of insulin (1 unit/kg) and glucose (3.2 g/kg) in an intraperitoneal injection. This ratio of insulin and glucose was predetermined in a series of test mice. The primary reason for not utilizing an intravenous infusion was the fact that the mechanical sensitivity measurement is an unrestricted behavior test and the presence of an intravenous access was felt to interfere with measurements.

### 2.5. CoQ10 Treatment

CoQ10 (Sigma-Aldrich, St. Louis, MO, USA) was dissolved in olive oil (Sigma-Aldrich) at a concentration of 30 mg/mL dosed at 100 mg/kg. This dose represents the human equivalent doses of 8 mg/kg, based on body surface area [9]. CoQ10 solution was prewarmed to 37°C and then injected intraperitoneally (i.p.) twice at a volume of 100 *µ*L/30 g of body weight before 20 hr and 4 hr of the induction of hypoglycemia.

### 2.6. Mechanical Allodynia Test

The mechanical allodynia test was conducted with a Touch-Test Sensory Evaluator (von Frey filaments, North Coast Medical, Inc., Wood Dale, IL, USA). Filaments were used to assess the degree of allodynia. For each assessment, the mouse was placed on a wire mesh platform and was covered with a transparent glass container and a period of 30 minutes was allowed for habituation. Five measurements were taken for each animal on the left paw. The observation of a positive response (paw lifting, “shaking,” or licking) within five seconds of the application of the filament was then followed by the application of a thinner filament (or a thicker one if the response was negative). The paw withdrawal threshold was measured five times and was expressed as the tolerance level in grams.

### 2.7. Immunohistochemistry and Image Quantification

Normal saline-injected control mice, mice with hypoglycemia induced by insulin, and hypoglycemic mice pretreated with CoQ10 were sacrificed via an overdose of Nembutal and were then decapitated. The L4-L5 lumber spinal cord was removed immediately. Part of the samples was fixed in 4% paraformaldehyde in phosphate buffered saline (pH 7.4) overnight, cryoprotected in 0.1 M phosphate buffered saline containing 20% sucrose, and sectioned by cryostat into 15 *μ*m thick sections. Sections were incubated overnight at 4°C with the primary antibody, anti-c-Fos (Sigma-Aldrich, USA), followed by biotinylated secondary antibody (Vector Lab, USA) for one hr at 22°C. To ensure the specificity of primary antibody, the primary antibody was replaced by the diluent of the antibody in one section in each set of stains so as to exclude nonspecific background staining.

Positive c-Fos cells were counted in laminar I-II area of 280 *µ*m^2^ of dorsal horn of lumbar spinal cord transverse section (the laminar I-II area is shown by the dotted line in Figure 4). The laminar I-II area size was calibrated using ImageJ software. Average cell numbers were counted from 6 sections of each group.

### 2.8. RNA Isolation and RT-PCR

The other half of the collected samples were fresh frozen in dry ice and stored at −80°C. The levels of mRNA of c-Fos were evaluated by RT-PCR in the DRG and spinal cord tissues. Extraction of total RNA was carried out with TRIzol (Invitrogen, Grand Island, NY, USA) according to the manufacturer's instructions. 1 *µ*g of RNA was reverse transcribed with 200 U/sample SuperScript II (Invitrogen) and 250 ng/reaction of random primers (Promega, San Luis Obispo, CA, USA). The genes of c-Fos were amplified from 0.1 *µ*g aliquots of cDNA in a standard PCR buffer (50 mM KCl, 1.5 mM MgCl_2_, and 10 mM Tris-HCl, pH 8.3) containing 10 pmol of forward and reverse primers along with 0.5 U/sample of AmpliTaq DNA polymerase (Applied Biosystems, Grand Island, NY, USA). Mouse *β*-actin was amplified as the internal control for the PCR reaction. The sequences of primer pairs are the following: *β*-actin forward: ctagacttcgagcaggagatg, reverse: caagaaggaaggctggaaaag, the product is 150 bp; c-Fos forward: ccagtcaagagcatcagcaa, reverse: aagtagtgcagcccggagta, the product is 247 bp.

### 2.9. Statistical Analysis

Data are presented as mean ± SEM and analyzed using Prism 4 software (GraphPad Software Inc., San Diego, CA). The behavior test data was analyzed with two-way analysis of variance with two repeated factors followed by Tukey's Multiple Comparison Test. Comparison between two groups was assessed by unpaired, two-tailed Student's *t*-test. *p* values < 0.05 were designated as statistically significant.

## 3. Results

### 3.1. The Effect of Glycemic Levels on Mechanical Sensitivity after Insulin or Saline Injection

**Figure 1:**
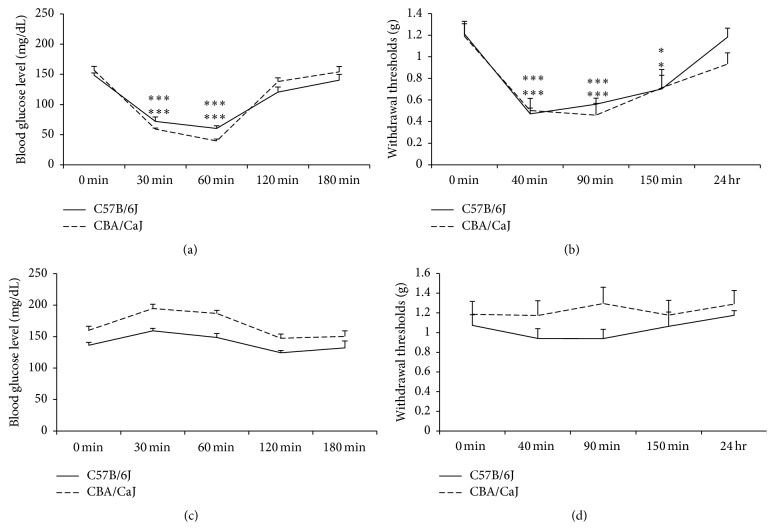
Effect of insulin treatment on glycemic levels and mechanical sensitivity. (a) Insulin injection decreases blood glucose levels at 30 and 60 min after insulin injection. (b) Mechanical sensitivity is increased (shown as decreased withdrawal thresholds) at 40, 90, and 150 min after insulin treatment. (c) Normal saline as control of insulin does not significantly affect blood glucose level or mechanical sensitivity (d). *∗* and *∗∗∗* represent *p* < 0.05 and *p* < 0.001, respectively, with ANOVA test.

Compared to control animals, it appeared that decreased blood glucose levels correlated to increased pain in the insulin treatment group. Both strains demonstrated significant differences in mechanical sensitivity 40, 90, and 150 min after insulin injection (*p* < 0.05 and *p* < 0.001).  Figure 1 shows that decreased withdrawal thresholds (mechanical hypersensitivity) were associated with insulin-induced acute hypoglycemia in both strains of mice. A group of normal saline-injected mice served as a control and demonstrated no changes in blood glucose levels or mechanical sensitivity, indicating that handling and injection stress did not affect or confound results.

### 3.2. The Effect of Combined Glucose and Insulin Injection on Mechanical Sensitivity

**Figure 2:**
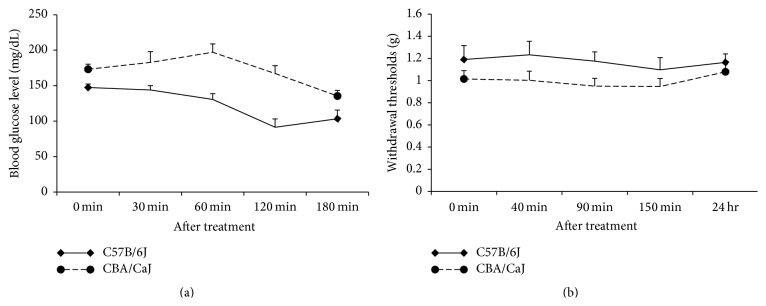
The effect of combined glucose and insulin injection on mechanical sensitivity. (a) demonstrates stable blood glucose levels after combined injection. (b) indicates that withdrawal thresholds showed no significant change after simultaneous injection of 1 unit/kg of insulin and 3.18 g/kg of glucose.

To determine whether insulin alone induces hypersensitivity, blood glucose levels were clamped at normal levels by joint insulin and glucose injection.  Table 1 demonstrated the blood glucose levels of two strains of mice in different situation: saline, insulin, or insulin combined with glucose. In the linked administration of insulin and glucose, blood glucose levels remained at an average of 123.33 ± 8.55 and 165.93 ± 10.60 mg/dL for the C57B/6J and CBA/CaJ mice, respectively, and these mice subsequently demonstrated no significant change in hindpaw withdrawal thresholds.  Figure 2 indicates that mechanical hypersensitivity did not develop when blood glucose levels remained in normal range after insulin was injected, suggesting that insulin itself is not involved in the hypoglycemia-induced mechanical hypersensitivity.

### 3.3. Pretreatment with CoQ10 Prevents the Development of Mechanical Hypersensitivity

**Figure 3:**
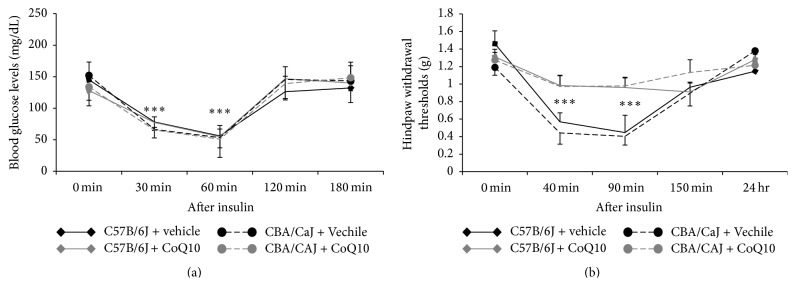
CoQ10 prevented the development of mechanical hypersensitivity. (a) CoQ10 had no effect on blood glucose levels. (b) In both strains tested, withdrawal thresholds remained stable with no significant changes in the group pretreated with CoQ10 compared to the prevehicle-injected group; *∗∗∗* represents *p* < 0.001 with ANOVA test.

CoQ10 has a critical role in producing energy and antioxidant protection for the body. For the scenario of insulin-induced hypoglycemia, we evaluated whether CoQ10 could play a protective role in the peripheral nerves.  Figure 3 indicates that CoQ10 did not affect the blood glucose level decrease following insulin injection; however, pretreatment with CoQ10 did prevent the development of mechanical hypersensitivity in insulin-induced hypoglycemic mice.

### 3.4. Hypoglycemia Induces Overexpression of c-Fos and CoQ10 Inhibits the Effect

**Figure 4:**
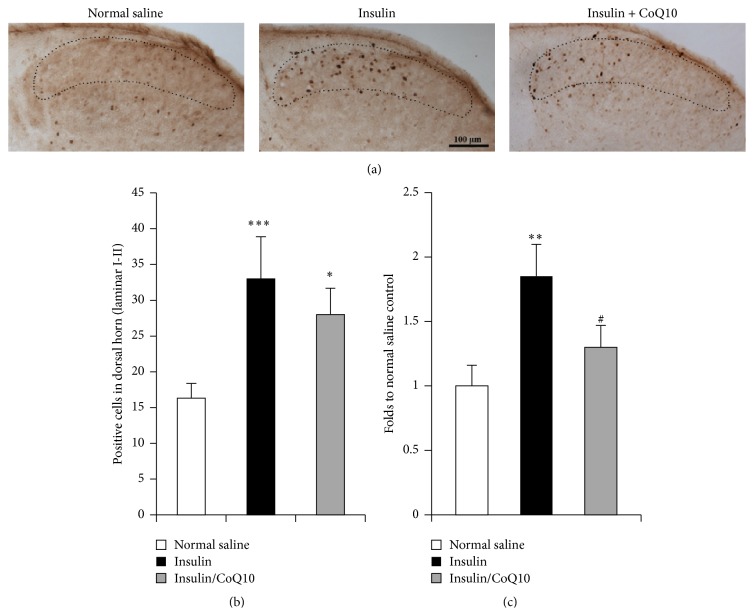
Immunohistology of c-Fos on lumber spinal cord and RT-PCR of c-Fos mRNA. (a) shows the expression of c-Fos in the dorsal horn of spinal cord of normal saline-injected mice, insulin-induced hypoglycemic mice, and insulin-induced hypoglycemic mice pretreated with CoQ10. (b) shows the results of image quantification in laminar I-II of the dorsal horn (dotted line area). (c) shows the result of quantification of RT-PCR of c-Fos mRNA. ^*∗∗∗*^ *p* < 0.001, ^*∗∗*^ *p* < 0.01, and ^*∗*^ *p* < 0.05 compared to normal saline controls; ^#^ *p* < 0.05, CoQ10 treated group compared to insulin group (Student's *t*-test).

c-Fos is a marker of acute stress in neuronal activation. Levels of c-Fos mRNA and c-Fos immunoreactivity within the spinal cord were evaluated in insulin-induced hypoglycemic mice. Figure 4 shows that c-Fos positive cells in the dorsal horn of the lumbar spinal cord after insulin injection increased significantly (in cell-counted analysis, positive cells in the insulin-injected group were more numerous than those in the saline-injected group, *p* < 0.01; in RT-PCR analysis, mRNA level of c-Fos in insulin-injected group is almost two times that in saline-injected group, *p* < 0.001; Student's *t*-test). However, pretreatment with CoQ10 partially decreased c-Fos expression in the spinal cord (in RT-PCR analysis, c-Fos mRNA levels in the group pretreated with CoQ10 were significantly lower than those in the insulin-injected group, *p* < 0.05).

## 4. Discussion

Studies have suggested that hypoglycemia-induced neuropathy may not simply be the result of glucose deprivation but rather a result of a multifactorial process involving oxidative stress and stress-sensitive factors [6]. The results of the present study demonstrate that insulin-induced hypoglycemia may result in acute neuropathic pain and the increased mechanical sensitivity noted is the result of decreased glycemic levels rather than insulin itself. The immunohistological and RT-PCR results suggest that insulin-induced hypoglycemia results in an increased expression of the stress-sensitive and pain-related factor c-Fos in nerve tissues. This in turn may be the mechanism by which acute pain is induced in the body. Furthermore, our results demonstrated that pretreatment with CoQ10 can prevent hypoglycemia-induced mechanical hypersensitivity and decrease the expression of c-Fos. Results further suggest that the protective effects of CoQ10 on pain sensitivity may be related to a decrease in activation of spinal pathways mediated by the inhibition of oxidative stress and intracellular signaling, preventing neuronal injury.

Patients with diabetes may face the difficult situation where tight blood glucose control can reduce the risk of diabetic complications; however, this degree of control may also increase the risk of dangerous hypoglycemic episodes. Studies estimate 30% of diabetics experience serious hypoglycemic episodes annually [10] and hypoglycemia has potentially devastating effects on nervous tissues. Clinicians have described acute severe painful neuropathy occurring during intensive treatment of patients with type 1 and type 2 diabetes treated with oral hypoglycemic agents or with insulin [1, 11]. In 1933, Caravati described neuropathic pain resulting from insulin use, “insulin neuritis”; however, the mechanism remains unclear [12].

Iatrogenic hypoglycemia continues to be a problem for patients with diabetes. Trophic factors and cytokines, including vascular endothelial growth factor (VEGF), insulin growth factor (IGF), mitogenic cytokine, IL-8, IL-6, and TNF-*α*, have been implicated in the pathogenesis of diabetic retinopathy [13], diabetic nephropathy [14], and diabetic neuropathy [15]. It is hypothesized that upregulation of these trophic factors and cytokines is associated with intensive glycemic control and is responsible for the early worsening of retinopathy and acute pain. Our data suggests that c-Fos, an immediate early transcription factor, is involved in insulin-induced hypersensitivity. Other researchers have observed an increase in proinflammatory cytokines in association with experimental hypoglycemia [16]. Elevated cytokine levels, including interleukin-1*β*, interleukin-6, and tumor necrosis factor-*α*, have been associated with impaired autonomic function after experimental hypoglycemia [17]. Thus, acute treatment of diabetes-induced neuropathy and retinopathy notably after intensive glycemic control may have a common pathophysiological mechanism that involves upregulation of proinflammatory cytokines. This concept also suggests an additional hypoglycemia-related pathophysiological mechanism and provides potential targets for therapeutic intervention.

Our data demonstrated that when combined, glucose and insulin injections, without subsequent hypoglycemic episodes, do not result in acute painful neuropathy, suggesting that insulin itself does not induce hypoglycemia-induced mechanical hypersensitivity. Thus, acute painful neuropathy is a concern not only for diabetics but also for normal subjects experiencing sudden hypoglycemic episodes. Therefore, “hypoglycemia-induced neuropathy” more accurately encompasses the disorder than the historic term, “insulin neuritis” [12]. Tight glucose control has been associated with numerous clinical benefits in diabetic patients, including the reduction of diabetic neuropathy; however, this type of treatment significantly increases the risk of severe hypoglycemic episodes. As we have demonstrated, hypoglycemia itself may exacerbate neuropathy and currently the only available method for preventing this hypoglycemia-induced neuronal injury in the clinical setting is the delivery of glucose, a treatment that paradoxically may exacerbate the insult. This study has obvious limitations; most notably, it was conducted solely in mice. It can be difficult to extrapolate data from lower mammals to humans; pain has many complex elements that can be difficult to assess.

Autophagy occurs in hypoglycemic peripheral nerves in association with axonal degeneration and regeneration in rats models [18]. Hypoglycemia causes Wallerian-type axonal degeneration of large myelinated nerve fibers in the peripheral nerve of insulin-treated diabetic animal models [19, 20]. Neuronal death resulting from hypoglycemia involves excitotoxicity and DNA damage [21]. By using cortical neuron cultures, researchers have found that application of poly(ADP-ribose) polymerase (PARP-1), an endogenous caspase-3 substrate inhibitor, increases neuronal survival in glucose deprivation. Additionally, rat models of insulin-induced hypoglycemia have shown the therapeutic potential of PAPD-1 inhibitors [21]. These results suggest that PARP-1 activation is a major factor mediating hypoglycemic neuronal death. Other researches have demonstrated that CoQ10 inhibits high glucose-induced cleavage of PAPD-1 [22] and suggest that CoQ10 prevents oxidative stress-induced apoptosis through inhibition of the mitochondria-dependent caspase-3 pathway. Taken together, our present results indicate that pretreatment with CoQ10 can prevent hypoglycemia-induced mechanical hypersensitivity and decrease the expression of c-Fos and chronic treatment with CoQ10 may scavenge free radicals instantly and prevent mitochondrial dysfunction in the transient hypoglycemia induced by tight glucose control in diabetics. This therapeutic approach may subsequently prevent nerve damage and suppress painful diabetic neuropathy.

## Figures and Tables

**Table 1:** Summary of blood glucose levels in different situation.

Strain	Treatment	Blood glucose level at different time point after treatment				
		0 min	30 min	60 min	120 min	180 min
C57B/6J	Saline	136.3 ± 4.69	159 ± 3.94	148.8 ± 6.14	124.3 ± 3.78	132.3 ± 10.86
	Insulin	148.4 ± 3.79	72^*∗∗∗*^ ± 7.37	160.2^*∗∗∗*^ ± 4.69	120.5 ± 8.40	140.2 ± 9.78
	Insulin + glucose	147.3 ± 4.98	144 ± 5.90	130.6 ± 7.99	103.3 ± 11.62	110 ± 12.23

CBA/CaJ	Saline	160 ± 6.55	194.6 ± 6.95	186.8 ± 5.06	147.5 ± 6.73	150.4 ± 8.77
	Insulin	165 ± 6.58	63.3^*∗∗∗*^ ± 1.76	47.25^*∗∗∗*^ ± 3.21	128.5 ± 6.24	189.6 ± 8.98
	Insulin + glucose	169.33 ± 7.33	175 ± 15.05	182.8 ± 11.63	167.1 ± 11.14	145 ± 7.85

^*∗∗∗*^ *p* < 0.001 compared to normal control in the same time point. ANOVA test.
